# Acupuncture treatment for insomnia based on the microbiome–gut–brain axis theory: A review

**DOI:** 10.1097/MD.0000000000046967

**Published:** 2026-01-02

**Authors:** Yipeng Gao, Xueping Yu

**Affiliations:** a Heilongjiang University of Chinese Medicine, Harbin, China; b The Third Ward of Acupuncture and Moxibustion Department, First Affiliated Hospital, Heilongjiang University of Chinese Medicine, Harbin, China.

**Keywords:** acupuncture treatment, insomnia, intestinal microbiota, mechanism, microbiome–gut–brain axis

## Abstract

Insomnia, a prevalent sleep disorder, significantly impacts patient social function and quality of life, creating a substantial burden on individuals. This underscores the need for effective treatments. Acupuncture, an essential part of complementary and alternative medicine, has received increasing attention for its therapeutic effect on insomnia, although its mechanism is still not fully understood. In recent years, some studies have focused on the microbiome–gut–brain axis, a promising area of study that may shed light on how acupuncture alleviates insomnia. This review explores the possible mechanisms by which acupuncture therapy improves insomnia through the microbiome–gut–brain axis. These mechanisms include adjusting the types and diversity of intestinal microbiome and altering short-chain fatty acid levels, inhibiting inflammatory responses, improving the tight connection of the intestinal mucosal barrier, controlling the release and production of brain–gut peptides, and regulating the pathways associated with the hypothalamic–pituitary–adrenal axis and the vagus nerve. The findings aim to provide a more objective basis for the use of acupuncture and moxibustion in treating insomnia.

## 1. Introduction

Insomnia is a sleep disorder characterized by difficulty falling asleep or staying asleep, resulting in poor sleep quality. This condition affects the optimal functioning of the human body and is often accompanied by daytime fatigue, lack of concentration, irritability, or apathy.^[1]^ A large body of research shows that the occurrence and development of various diseases, including obesity, cardiovascular disease, psychiatric disease, and diabetes, are closely entwined with insomnia.^[2–5]^ Additionally, chronic insomnia can increase the risk of mortality.^[6]^ Epidemiological surveys estimate that ~19% to 50% of individuals worldwide experience symptoms of insomnia, showing a progressively increasing trend.^[7]^

Acupuncture is a therapeutic approach rooted in traditional Chinese medicine (TCM), characterized by its ease of application, affordability, and patient acceptance.^[8]^ Compared with hypnotics and sedatives, acupuncture offers the benefits of high safety and absence of drug dependence for treating insomnia.^[9]^ Additionally, acupuncture has a good therapeutic effect on anxiety and depression symptoms complicated by insomnia.^[10]^ Unlike Western medicines that only focus on insomnia symptoms, personalized acupuncture therapy can increase or decrease acupuncture points based on the patient’s unique symptoms, thereby enhancing treatment results.^[11]^ Consequently, acupuncture has become the preferred treatment for many individuals suffering from insomnia. However, the mechanism of acupuncture in the treatment of insomnia remains incompletely understood, necessitating further research to refine its theory.

**Figure 1.**
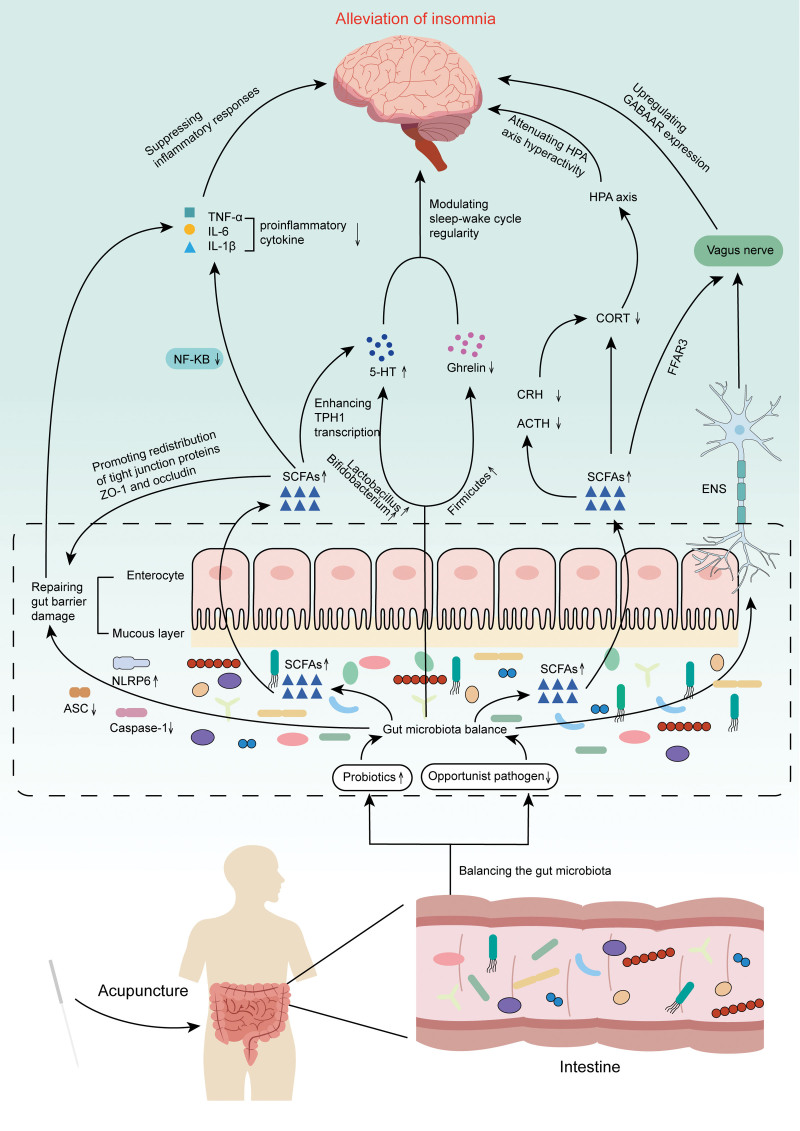
The mechanism of acupuncture in treating insomnia by adjusting the intestinal microbiota. Acupuncture treatment can inhibit inflammation, improve the tight connectivity of the intestinal mucosal barrier, adjust the release and production of brain–gut peptides, regulate hypothalamus–pituitary–adrenal axis-related pathways, and control the vagus nerve pathway, thereby alleviating insomnia symptoms.

In recent years, research into the correlation between intestinal microbiota and insomnia has deepened, making the theory of the regulation of insomnia through microbiome–gut–brain (MGB) axis a new research hotspot. This review systematically explores the mechanism of acupuncture in treating insomnia by adjusting the intestinal microbiota (Fig. 1), providing a new objective basis for acupuncture’s efficiency in insomnia treatment.

## 2. Literature search and selection

We conducted a systematic search across PubMed, Web of Science, Embase, and China National Knowledge Infrastructure databases using the following combined keywords: “microbiota–gut–brain (MGB) axis,” “insomnia,” “acupuncture therapy,” “gut microbiota,” “short-chain fatty acids (SCFAs),” “intestinal mucosal barrier,” “immune inflammation,” “brain-gut peptides,” “serotonin,” “ghrelin,” “hypothalamic–pituitary–adrenal (HPA) axis,” “vagus nerve,” and “enteric nervous system.” The literature search focused on articles published from January 2000 to August 2024, with supplementary inclusion of manually screened reference lists. Inclusion criteria mandated relevance to at least one of the following topics: acupuncture intervention, insomnia improvement, or MGB axis mechanisms. Studies lacking control groups, incomplete microbiota analyses, or case reports were excluded, ensuring methodological rigor in literature selection.

## 3. Correlation between MGB axis and insomnia

### 3.1. MGB axis

The gut microbiome, also known as intestinal microbiota, refers to the normal microorganisms that colonize the human gastrointestinal tract and coexist in symbiosis with more than 10^13^ to 10^18^ intestinal microbes in humans.^[12]^ Among them, *Firmicutes* and *Bacteroidetes* are the most numerous, accounting for ~92% of the human microbiome.^[13]^ The gut microbiota play a key role in coordinating brain development and behavior,^[14]^ whereas the brain can also adjust the structure and diversity of the gut microbiota,^[15]^ creating a bidirectional regulatory channel known as the MGB axis. Studies have shown that the gut microbiota regulates brain function through 4 main pathways that facilitate this bidirectional information flow.

The first pathway is dominated by metabolites of the gut microbiota, particularly short-chain fatty acids (SCFAs), which participate in the vagus nerve, immune, and neuroendocrine pathways. First, SCFAs can reverse microglia damage^[16]^; they also regulate the production of brain-derived neurotrophic factor, which signals the brain via the vagus nerve.^[17]^ Second, SCFAs can mediate the immune response by regulating the size and function of regulatory T-cells, thereby influencing brain function.^[18]^ Third, SCFAs play a vital part in the production and release of 5-hydroxytryptamine (5-HT), which has extensive projections to the brain and regulates important functions such as sleep and mood.^[19]^

The second pathway involves the immune system, where the gut microbiota is essential for inhibiting pathogenic bacterial colonization and protecting the intestinal barrier.^[20]^ When the gut microbiota is disrupted, intestinal permeability increases, leading to translocation of gram-negative bacteria containing lipopolysaccharide that can overactivate the immune system,^[16]^ resulting in elevated concentrations of pro-inflammatory cytokines and damage to central nervous system (CNS) cells.^[21]^ Additionally, the diversity of the gut microbiota directly affects the maturation and activation of microglia in the CNS.^[22]^

The third is the neuroendocrine pathway, in which gut microbiota serves as the main producer of neurotransmitters.^[23]^ These neurotransmitters affect their production and synthesis by regulating the metabolism and content of amino acids in certain intestinal endocrine cells and neurotransmitter precursors. Excessive or insufficient production of these neurotransmitters can affect the CNS function.^[24]^ Additionally, gut microbiota can influence the hypothalamic–pituitary–adrenal (HPA) axis by regulating corticotropin-releasing factor and cortisol levels,^[21]^ making the HPA axis a key factor in mental disorders such as anxiety, depression, and insomnia.^[25]^

The fourth is the vagus nerve pathway. In this pathway, the vagus afferent nerve reaches the mucosal and intestinal muscle layer near enteroendocrine cells, mucosal immune cells, and neurons of the enteric nervous system (ENS). These structures form synapses with intestinal epithelial cells and enteroendocrine cells, rapidly conducting signals from the intestinal lumen to the vagus nerve through glutamatergic and serotonergic pathways. The cell bodies of the vagus afferent nerves reside in the nodular ganglia, where vagus nerve fibers project to the nucleus of the solitary tract, area postrema, and dorsal nucleus of the vagus nerve. This information transmission pathway is referred to as the gut microbiome–ENS–vagus–brain pathway.^[26,27]^

### 3.2. Gut microbiota and insomnia

Modern research has indicated that the gut microbiota regulates the host’s circadian rhythm in various ways, and it is important for etiology and pathogenesis of insomnia.^[28]^ Gut-derived neurotransmitters (such as 5-HT and gamma-aminobutyric acid) can directly affect the brain and interact with the CNS.^[29,30]^ Notably, these neurotransmitters can interact with afferent neurons of the vagus nerve, influencing the neural circuits involved in sleep–wake regulation, thereby altering sleep–wake structure.^[31]^ Meanwhile, gut microbiota metabolites such as SCFAs play a vital part in improving sleep and regulating circadian rhythms. Szentirmai et al found that butyrate can induce an increase in non-rapid eye movement sleep in mice, suggesting that certain receptors or specific pathways within the hepatic portal vein can promote sleep through the action of butyrate.^[32]^ In addition, intestinal immune function is a key factor affecting sleep. Several studies have shown that pro-inflammatory cytokines can influence sleep structures.^[33–35]^ When the balance of the gut microbiota is broken, the intestinal barrier function is impaired, triggering an inflammatory response and promoting the production of pro-inflammatory cytokines.^[36]^ Take together, the gut microbiota and its metabolites play an important part in the MGB axis and are indispensable in the pathophysiology of intestinal homeostasis and sleep.

## 4. TCM theory analysis of MGB axis and insomnia

In exploring the pathogenesis of insomnia, TCM emphasizes the relationships between the gastrointestinal tract and the brain. The brain is considered the residence of the original spirit. The concept that “stomach disharmony leads to restless sleep” dates back to the Su Wen, aligning with the modern theory of the MGB axis. Here, “stomach” is not limited to its modern definition; it encompasses the large intestine, small intestine, spleen, stomach, and Yangming meridian. As stated in the Miraculous Pivot: “The large intestine and small intestine all belong to the stomach, and they are the foot Yangming stomach channel.^[37]^” The following analysis will explore the relationship between “stomach disharmony” and “restless sleep” from 2 perspectives.

The meridians can connect upward and downward, communicate inside and outside, and serve as the channels for the flow of qi and blood in the human body.^[38]^ According to the Miraculous Pivot: “The foot Yangming stomach channel, starting from the radix nasi, follows the hairline to the point where the forehead meets the skull.” This indicates that the foot Yangming stomach channel can extend to the brain. The “Revised Popular Guide to ‘Treatise on Cold Damage’” states that “The branches of the stomach meridian extend to the heart and brain. When an evil fire obstructs these pathways, it blocks the orifices of the spirit, resulting in confusion and delirium.” Pathologically, when the Yangming meridian is filled with qi and blood and evil heat accumulates in the stomach, it disturbs the meridians and obscures the clarity of the mind, potentially leading to insomnia. This demonstrates the close relationship between the brain and the “stomach” through the meridians.

The spleen is the foundation of acquired constitution and the source of qi and blood production, regulating qi movement in the body. The small intestine separates the clear from the turbid, whereas the large intestine transmits dross. Altogether, the spleen and stomach metabolize human fluids. As stated in Dong-yuan’s Proven Formulas: “The stomach and intestines are rewound, but the qi cannot be transformed.” If the stomach and intestines are impaired, the body’s qi movement becomes disordered, leading to abnormal fluid metabolism and the accumulation of phlegm-rheum. Phlegm-rheum obstructs the flow and transforms into heat, which disturbs the spirit and causes insomnia. Additionally, dysfunction of the spleen and stomach prevents the clear yang from ascending and the turbid yin from descending, disrupting the harmony of the 9 orifices and resulting in a restless mind that affects sleep.

## 5. Acupuncture mechanism in treating insomnia by modulating the MGB axis

Acupuncture is an effective treatment for insomnia. A recent randomized controlled trial demonstrated that acupuncture had a sleep-promoting effect in the short to medium term.^[39]^ Zhao et al conducted a meta-analysis showing that acupuncture treatment, when administered at least 12 times, significantly improved sleep efficiency and duration in patients with insomnia.^[40]^ Recently, the development of the MGB axis theory has led to new insights into the mechanisms by which acupuncture treats insomnia. The following sections will elaborate on the potential mechanisms of acupuncture in treating insomnia based on the MGB axis theory from 4 perspectives.

### 5.1. Acupuncture regulates gut microbiota and SCFAs

Modern studies indicate that acupuncture can adjust the ecological stability of gut microbiota and balance the number and proportion of probiotics and pathogens in the host.^[41]^ Zhang et al performed electroacupuncture intervention in mice for 2 weeks and found that electroacupuncture increased the abundance of probiotics, such as Brault bacteria and *Lactobacillus*, whereas reducing opportunistic pathogens such as *Prevotella* and *Helicobacter*.^[42]^ Two recent studies suggest that increased abundance of *Bacteroidetes* may serve as a biomarker for identifying insomnia.^[34,43]^ Additionally, another study found that the relative abundance of lactobacilli in the left fusiform gyrus in patients with chronic insomnia was significantly negatively correlated with regional homogeneity, suggesting that gut microbiota can influence brain function.^[44]^ Hong et al stimulated insomnia in mice through acupuncture at Baihui (DU20), Sanyinjiao (SP6), and Shenmen (HT7), reporting improved sleep status, a decrease in intestinal Bacteroides, and increase in *Lactobacillus*.^[45]^ This led to the conclusion that acupuncture may improve the sleep–wake cycle by regulating intestinal microbiota abundance.

Gut microbiota metabolites, such as SCFAs, have been shown to affect sleep physiology by regulating inflammatory factor levels, downregulating HPA axis reactivity, promoting the secretion and release of 5-HT, and activating vagus nerve receptors.^[27,46–49]^ SCFAs secreted by probiotics such as *Enterobacter faecalis* and *Agassobacter* in the gut can inhibit intestinal colonization by opportunistic pathogens and reduce inflammatory factor production.^[50]^ Ouyang et al performed moxibustion on the Guanyuan (RN4) in elderly rats for 40 days, finding that the relative abundance of lactobacilli increased, consequently raising the levels of SCFAs in the intestine.^[51]^ Ke et al found that electroacupuncture increased acetic acid levels, which in turn reduced intestinal inflammation.^[52]^ These studies collectively showed that acupuncture could regulate SCFA levels in the gut.

In summary, acupuncture treatment effectively improves insomnia, potentially by regulating intestinal microbiota and SCFA levels and thereby improving the MGB axis pathway.

### 5.2. Acupuncture regulates the intestinal mucosal barrier and immune inflammation

Modern research has found that inflammation plays a significant role in the pathogenesis of metabolic and neurodegenerative diseases associated with sleep deprivation^[53,54]^ and is closely related to the intestinal mucosal barrier.^[55]^ Typically, the gut microbiota regulates this barrier. When exogenous pathogenic microorganisms invade, probiotics such as bifidobacteria interact with intestinal mucosal cells to form a biological barrier that protects the intestinal microecosystem.^[56]^ However, when the balance of the intestinal microbiota is disrupted, the intestinal barrier is compromised, allowing microbiota metabolites (such as lipopolysaccharide) to enter the circulatory system. These metabolites interact with endotoxin-binding proteins and aberrantly activate the Toll-like receptor 4/myeloid differentiation factor 88/nuclear factor kappa-B pathway, inducing inflammatory responses and promoting the production of pro-inflammatory cytokines such as TNF-α, IL-6, and IL-1β.^[36,57]^ Additionally, pro-inflammatory cytokines can further damage tight junctions and increase intestinal permeability.^[58]^ Cai et al measured serum levels of diamine oxidase, D-lactate, intestinal fatty acid-binding protein, and endothelin in 45 patients with chronic insomnia and 30 healthy volunteers, finding that these intestinal epithelial barrier markers were significantly positively correlated with sleep efficiency.^[59]^ They hypothesized that the occurrence and progression of insomnia disorder may be related to intestinal barrier damage. Therefore, regulating the intestinal flora, repairing the intestinal mucosal barrier, and improving inflammation are essential for treating insomnia.

Acupuncture effectively regulates intestinal microbiota, repairs the intestinal mucosal barrier, and improves the inflammatory response.^[60–63]^ Bao et al found that acupuncture helped restore the balance of the gut microbiota and increased the abundance of anti-inflammatory bacteria and bacteria that produce SCFAs, thereby enhancing gut barrier integrity and inhibiting the inflammatory response.^[64]^ Bao et al performed moxibustion treatment on rats at bilateral Tianshu (ST25) and Zusanli (ST36) for 7 consecutive days, and finding a significant increase in the relative DNA abundance of *Lactobacillus* and *Bifidobacterium* in the intestinal tract, a decrease in *Escherichia coli*, an increase in the expression of NOD-like receptor family pyrin domain containing 6, and a decrease in the expression of apoptosis-associated Speck-like protein containing a CARD and cysteine-requiring aspartate protease-1.^[65]^ Transmission electron microscopy images showed that moxibustion alleviated mucosal injury in the colon. These findings suggest that moxibustion can balance gut microbiota, improve intestinal inflammation, and alleviate mucosal damage. In a recent study, electroacupuncture at Zusanli (ST36) and Kunlun (BL60) inhibited the expression of Toll-like receptor 4, MYD88, and nuclear factor kappa-B, thereby improving the inflammatory response.^[66]^ In addition, electroacupuncture at Zusanli (ST36) can attenuate the distortion of intestinal glial cells and increase the expression of zonula occludens-1 protein, thereby maintaining intestinal barrier function and reducing intestinal permeability.^[67]^ In summary, acupuncture can improve the MGB axis pathway, potentially by regulating the intestinal flora, inhibiting the inflammatory response, and improving the tight connectivity of the intestinal mucosal barrier, offering therapeutic benefits for patients with insomnia.

### 5.3. Acupuncture regulates neuroendocrine pathways

#### 5.3.1. Acupuncture regulates the release and production of brain–gut peptides

Brain–gut peptides are bioactive peptides distributed in the brain and gastrointestinal tract. They can facilitate bidirectional information transmission and play a huge part in regulating the functional activities of the gastrointestinal tract and CNS.^[68]^ Modern studies have shown that changes in the content of brain–gut peptides, such as 5-HT and growth hormone releasing peptide (ghrelin), affect patients’ sleep–wake cycle,^[69,70]^ and the secretion and synthesis of these brain–gut peptides are regulated by the gut microbiota.^[19,71]^

5-HT is one of the first substances found as a participating factor in the adjustment of sleep–wake structures, playing a crucial role in the preparation, triggering, and maintenance of sleep.^[72]^ Physiological studies have demonstrated that 5-HT induces sleep by inhibiting the midbrain reticular activation system or the locus coeruleus–norepinephrine system.^[73]^ Additionally, early research indicates that probiotics, such as *Lactobacillus* and *Bifidobacterium*, can enhance tryptophan metabolism and support the direct conversion of tryptophan to 5-HT.^[74]^ Ogawa et al found in animal studies that dysregulation of gut microbiota leads to a sharp decline in 5-HT levels, thereby affecting the sleep–wake cycle.^[75]^ Therefore, 5-HT may participate in the communication between gut microbiota and the sleep regulatory system. Studies have shown that electroacupuncture stimulation at Baihui (GV20) and Zusanli (ST36) can increase the abundance of probiotics such as *Lactobacillus* and *Bifidobacterium*,^[76]^ whereas acupuncture can increase tryptophan content in the peripheral system, elevate the level of 5-HT in the blood, and accelerate the transport of tryptophan in the blood, thereby promoting the synthesis of 5-HT.^[77]^

Ghrelin is a multifunctional brain–gut peptide hormone that is widely expressed in the cerebral cortex, hypothalamus, hippocampus, striatum, and other regions. It plays an important part in multifaceted brain functions such as sleep, memory, and neuroprotection.^[78,79]^ Ghrelin induces arousal and inhibits non-rapid eye movement sleep and rapid eye movement sleep.^[80]^ Szentirmai et al injected ghrelin into the ventricles and hypothalamus of rats, demonstrating a strong arousal effect.^[81]^ Another study found an inverse correction between firmicutes and ghrelin levels in humans and rodents, suggesting that ghrelin may be integral to the bidirectional communication between gut microbiota and the sleep regulatory system.^[82,83]^ Concurrently, Ouyang et al reported a significant increase in the abundance of Firmicutes in the gut of mice after 40 days of moxibustion on Guanyuan (RN4).^[51]^ In addition, acupuncture and thermal moxibustion can reduce ghrelin levels in mice.^[84]^

Therefore, acupuncture can improve sleep, possibly by regulating gut microbiota composition, facilitating brain–gut interactions, promoting the release of 5-HT, and inhibiting ghrelin production.

#### 5.3.2. Acupuncture inhibits hyperactivity of the HPA axis

The HPA axis involves the neuroendocrine system and plays an important part in the MGB axis.^[85,86]^ It is influenced by circadian oscillators, which regulate sleep and maintain bioalertness.^[87]^ An overactive HPA axis impairs sleep quality by causing sleep fragmentation, decreased slow-wave sleep, and shortened sleep duration, which is thought to be induced by increased plasma levels of corticotropin-releasing hormone (CRH) and cortisol (CORT).^[88,89]^ Acupuncture can ameliorate sleep by regulating HPA axis-related pathways. Some studies have indicated that acupuncture can downregulate the expression of CRH and adrenocorticotropic hormone in the hypothalamus and pituitary gland, thereby reducing CORT release.^[90]^ This hypothesis has been demonstrated in several studies.^[91,92]^ Xie et al found that rats with insomnia have improved sleep and restored abnormally elevated levels of hypothalamic corticosterone, adrenocorticotropic hormone, and CRH after electroacupuncture.^[93]^ In addition, Lv et al confirmed that abundance differences in gut microbiota are closely related to the expression of HPA axis-related factors through correlation analysis.^[94]^ If the gut microbiota is out of balance, the stage generation of corticosterone in the ileum will be affected, resulting in a persistent increase in CORT.^[95]^ Probiotic pretreatment in rats has also been shown to reduce the hyperresponsiveness of the HPA axis.^[96]^ Acupuncture is able to reverse the imbalance of the gut microbiota and increase the abundance of probiotics in the gut. Xie et al performed electroacupuncture treatment on Zusanli (ST36) and Yanglingquan (GB34) in mice for 2 weeks and found that the abundance of probiotics in the intestine is increased.^[97]^ Furthermore, electroacupuncture can reduce the ratio of Firmicutes to Bacteroidetes, which is often considered a relative measure of the balance or dysbiosis of the gut microbiota, suggesting that electroacupuncture can reverse imbalances in gut microbiota.^[98]^ Therefore, acupuncture can achieve therapeutic effects on patients with insomnia, possibly by regulating the intestinal microbiota, adjusting the content of HPA axis-related factors, and improving the MGB axis pathway.

### 5.4. Acupuncture regulates the vagus nerve pathway

The vagus nerve is a brain–gut information pathway between the CNS and ENS. Gut microbes and their metabolites can affect neurons in the ENS and interact with the afferent pathway of the vagus nerve, thereby affecting the neural circuits involved in sleep–wake regulation.^[99]^ Probiotics have been found to modulate vagus nerve-dependent pathways, which in turn increase the expression of central γ-aminobutyric acid A-type receptors (GABAAR) in mice.^[100]^ However, these effects can be blocked by vagotomy, suggesting that GABAAR and the vagus nerve are jointly involved in the 2-way exchange of information between gut microbiota and the brain’s sleep regulation system.^[101]^

Acupuncture has been shown to modulate vagus nerve activity and increase GABAAR expression. A recent study showed that electroacupuncture stimulates the vagus nerve and acts on GABAAR to improve sleep.^[102]^ Furthermore, electroacupuncture reportedly activates the GABAergic system, increases GABAAR expression and activity, enhances neurosuppressive effects, and improves sleep quality.^[103,104]^ In addition, acupuncture has been shown to increase the DNA abundance of intestinal probiotics. Xiao et al found that the DNA abundance in probiotics such as *Lactobacillus* and *Bifidobacterium* was significantly increased by electroacupuncture treatment at Baihui (DU20) and Zusanli (ST36) in mice.^[105]^

Therefore, acupuncture may prolong sleep duration and improve sleep conditions by increasing the abundance and diversity of intestinal probiotics, regulating vagus nerve pathways, and increasing GABAAR expression.

## 6. Research limitations and future directions

While significant progress has been achieved in this field, current research efforts remain constrained by some limitations. First, investigations remain disproportionately focused on preclinical animal models, with insufficient clinical trials validating therapeutic mechanisms in human populations. Most studies inadequately address the ecological dynamics of human gut microbiota, particularly their spatiotemporal interactions with environmental confounders that substantially shape microbial community architecture. Furthermore, translational gaps persist between mechanistic insights into host–microbe crosstalk and their clinical applications in precision medicine. Consequently, applying multi-omics technologies in clinical research holds significant value.

Second, although accumulating evidence indicates that acupuncture may alleviate insomnia through modulation of the MGB axis, the precise effector pathways and molecular targets governing this neuromicrobial interplay remain uncharacterized. Notably, critical gaps persist in direct neurobiological validation of the causal chain linking microbial modulation to cerebral functional reorganization. To address these limitations, future investigations should integrate functional magnetic resonance imaging and metabolomics to longitudinally assess gut microbiota dynamics and neural pathway alterations during acupuncture interventions.

Finally, while preliminary clinical evidence supports short-term efficacy, the long-lasting therapeutic effects of acupuncture remain inconclusive due to insufficient long-term follow-up data. Furthermore, significant methodological heterogeneity in subject selection criteria and acupoint combination protocols may compromise the comparability of research outcomes. Future investigations should establish standardized acupoint selection guidelines, implement multicenter randomized controlled trials with large cohorts, and extend follow-up durations to strengthen the evidence base.

## 7. Conclusion

The MGB axis plays an important part in the onset and progression of insomnia. Currently, the main pathways identified to improve insomnia include the immune, neuroendocrine, vagus nerve, and bidirectional information transmission pathways dominated by gut microbiota metabolites. Existing evidence indicates that acupuncture treatment can inhibit inflammation, improve the tight connectivity of the intestinal mucosal barrier, adjust the release and production of brain–gut peptides, regulate HPA axis-related pathways, and control the vagus nerve pathway, thereby alleviating insomnia symptoms.

This review highlights the possible mechanism by which acupuncture therapy regulates the MGB axis to improve insomnia and provides modern theoretical support for acupuncture in the treatment of insomnia. We anticipate that with advancing research, the therapeutic role of the MGB axis in insomnia will be comprehensively elucidated, thereby accelerating the modernization of traditional medical practices and providing novel objective evidence for acupuncture in insomnia management.

## Author contributions

**Conceptualization:** Yipeng Gao.

**Methodology:** Yipeng Gao.

**Visualization:** Yipeng Gao.

**Writing – original draft:** Yipeng Gao.

**Writing – review & editing:** Yipeng Gao, Xueping Yu.

## References

[1] Riemann D Espie CA Altena E. The European insomnia guideline: an update on the diagnosis and treatment of insomnia 2023. J Sleep Res. 2023;32:e14035. 38016484 10.1111/jsr.14035

[2] Zhou L Kong J Li X Ren Q. Sex differences in the effects of sleep disorders on cognitive dysfunction. Neurosci Biobehav Rev. 2023;146:105067. 36716906 10.1016/j.neubiorev.2023.105067

[3] Berger M Solelhac G Roche F Heinzer R. Insomnia, a new modifiable risk factor for heart failure? Eur Heart J. 2021;42:4177–9. 34417611 10.1093/eurheartj/ehab570

[4] Gangitano E Martinez-Sanchez N Bellini MI. Weight loss and sleep, current evidence in animal models and humans. Nutrients. 2023;15:3431. 37571368 10.3390/nu15153431 PMC10420950

[5] Schipper SBJ Van Veen MM Elders PJM. Sleep disorders in people with type 2 diabetes and associated health outcomes: a review of the literature. Diabetologia. 2021;64:2367–77. 34401953 10.1007/s00125-021-05541-0 PMC8494668

[6] von Schantz M Ong JC Knutson KL. Associations between sleep disturbances, diabetes and mortality in the UK Biobank cohort: a prospective population-based study. J Sleep Res. 2021;30:e13392. 34101927 10.1111/jsr.13392 PMC8612946

[7] Krystal AD Ashbrook LH Prather AA. What is insomnia? JAMA. 2021;326:2444. 34932076 10.1001/jama.2021.19283

[8] Lin YF Liu ZD Ma W Shen WD. Hazards of insomnia and the effects of acupuncture treatment on insomnia. J Integr Med. 2016;14:174–86. 27181124 10.1016/S2095-4964(16)60248-0

[9] Zhao FY Fu QQ Spencer SJ. Acupuncture: a promising approach for comorbid depression and insomnia in perimenopause. Nat Sci Sleep. 2021;13:1823–63. 34675729 10.2147/NSS.S332474 PMC8520448

[10] Chen Z Jiang T Yin X Li B Tan Z Guo J. The increased functional connectivity between the locus coeruleus and supramarginal gyrus in insomnia disorder with acupuncture modulation. Front Neurosci. 2023;17:1131916. 37152608 10.3389/fnins.2023.1131916 PMC10157050

[11] Son C Lim YC Lee YS Lim JH Kim BK Ha IH. Analysis of medical services for insomnia in Korea: a retrospective, cross-sectional study using the health insurance review and assessment claims data. Healthcare (Basel). 2021;10:7. 35052172 10.3390/healthcare10010007 PMC8775632

[12] Anand N Gorantla VR Chidambaram SB. The role of gut dysbiosis in the pathophysiology of neuropsychiatric disorders. Cells. 2022;12:54. 36611848 10.3390/cells12010054 PMC9818777

[13] Shi N Li N Duan X Niu H. Interaction between the gut microbiome and mucosal immune system. Mil Med Res. 2017;4:14. 28465831 10.1186/s40779-017-0122-9 PMC5408367

[14] Fung TC Olson CA Hsiao EY. Interactions between the microbiota, immune and nervous systems in health and disease. Nat Neurosci. 2017;20:145–55. 28092661 10.1038/nn.4476 PMC6960010

[15] Vanuytsel T van Wanrooy S Vanheel H. Psychological stress and corticotropin-releasing hormone increase intestinal permeability in humans by a mast cell-dependent mechanism. Gut. 2014;63:1293–9. 24153250 10.1136/gutjnl-2013-305690

[16] Horn J Mayer DE Chen S Mayer EA. Role of diet and its effects on the gut microbiome in the pathophysiology of mental disorders. Transl Psychiatry. 2022;12:164. 35443740 10.1038/s41398-022-01922-0 PMC9021202

[17] Wang Y Du W Hu X. Targeting the blood-brain barrier to delay aging-accompanied neurological diseases by modulating gut microbiota, circadian rhythms, and their interplays. Acta Pharm Sin B. 2023;13:4667–87. 38045038 10.1016/j.apsb.2023.08.009 PMC10692395

[18] Wang J Zhu N Su X Gao Y Yang R. Gut-microbiota-derived metabolites maintain gut and systemic immune homeostasis. Cells. 2023;12:793. 36899929 10.3390/cells12050793 PMC10000530

[19] Yano JM Yu K Donaldson GP. Indigenous bacteria from the gut microbiota regulate host serotonin biosynthesis. Cell. 2015;161:264–76. 25860609 10.1016/j.cell.2015.02.047 PMC4393509

[20] Zhou B Yuan Y Zhang S. Intestinal flora and disease mutually shape the regional immune system in the intestinal tract. Front Immunol. 2020;11:575. 32318067 10.3389/fimmu.2020.00575 PMC7147503

[21] Góralczyk-Bińkowska A Szmajda-Krygier D Kozłowska E. The microbiota-gut-brain axis in psychiatric disorders. Int J Mol Sci. 2022;23:11245. 36232548 10.3390/ijms231911245 PMC9570195

[22] Cryan JF Dinan TG. Gut microbiota: microbiota and neuroimmune signalling-Metchnikoff to microglia. Nat Rev Gastroenterol Hepatol. 2015;12:494–6. 26215386 10.1038/nrgastro.2015.127

[23] Smith PA. The tantalizing links between gut microbes and the brain. Nature. 2015;526:312–4. 26469024 10.1038/526312a

[24] Ganci M Suleyman E Butt H Ball M. The role of the brain-gut-microbiota axis in psychology: the importance of considering gut microbiota in the development, perpetuation, and treatment of psychological disorders. Brain Behav. 2019;9:e01408. 31568686 10.1002/brb3.1408 PMC6851798

[25] Mhanna A Martini N Hmaydoosh G. The correlation between gut microbiota and both neurotransmitters and mental disorders: a narrative review. Medicine (Baltimore). 2024;103:e37114. 38306525 10.1097/MD.0000000000037114 PMC10843545

[26] Longo S Rizza S Federici M. Microbiota-gut-brain axis: relationships among the vagus nerve, gut microbiota, obesity, and diabetes. Acta Diabetol. 2023;60:1007–17. 37058160 10.1007/s00592-023-02088-x PMC10289935

[27] Dowling LR Strazzari MR Keely S Kaiko GE. Enteric nervous system and intestinal epithelial regulation of the gut-brain axis. J Allergy Clin Immunol. 2022;150:513–22. 36075637 10.1016/j.jaci.2022.07.015

[28] Song D Yang CS Zhang X Wang Y. The relationship between host circadian rhythms and intestinal microbiota: a new cue to improve health by tea polyphenols. Crit Rev Food Sci Nutr. 2021;61:139–48. 31997655 10.1080/10408398.2020.1719473

[29] Byun JI Shin YY Chung SE Shin WC. Safety and efficacy of gamma-aminobutyric acid from fermented rice germ in patients with insomnia symptoms: a randomized, double-blind trial. J Clin Neurol. 2018;14:291–5. 29856155 10.3988/jcn.2018.14.3.291 PMC6031986

[30] Feng W Yang Z Liu Y. Gut microbiota: a new target of traditional Chinese medicine for insomnia. Biomed Pharmacother. 2023;160:114344. 36738504 10.1016/j.biopha.2023.114344

[31] Hou X Rong C Wang F Liu X Sun Y Zhang HT. GABAergic system in stress: implications of gabaergic neuron subpopulations and the gut-vagus-brain pathway. Neural Plast. 2020;2020:8858415. 32802040 10.1155/2020/8858415 PMC7416252

[32] Szentirmai E Millican NS Massie AR Kapás L. Butyrate, a metabolite of intestinal bacteria, enhances sleep. Sci Rep. 2019;9:7035. 31065013 10.1038/s41598-019-43502-1 PMC6504874

[33] Hogan D Morrow JD Smith EM Opp MR. Interleukin-6 alters sleep of rats. J Neuroimmunol. 2003;137:59–66. 12667648 10.1016/s0165-5728(03)00038-9

[34] Li Y Zhang B Zhou Y. Gut microbiota changes and their relationship with inflammation in patients with acute and chronic insomnia. Nat Sci Sleep. 2020;12:895–905. 33177907 10.2147/NSS.S271927 PMC7652227

[35] Smith RP Easson C Lyle SM. Gut microbiome diversity is associated with sleep physiology in humans. PLoS One. 2019;14:e0222394. 31589627 10.1371/journal.pone.0222394 PMC6779243

[36] Wang JW Pan YB Cao YQ. Loganin alleviates LPS-activated intestinal epithelial inflammation by regulating TLR4/NF-κB and JAK/STAT3 signaling pathways. Kaohsiung J Med Sci. 2020;36:257–64. 31859422 10.1002/kjm2.12160 PMC11896463

[37] Ju K Shi Q Guan R. Study on the mechanism of Jiao-Tai-Wan in treating primary insomnia based on intestinal microbiota. J Psychiatry. 2022;35:543–7.

[38] Ye X Ren YL Chen YH Chen J Tang XJ Zhang ZM. A “4D” systemic view on meridian essence: substantial, functional, chronological and cultural attributes. J Integr Med. 2022;20:96–103. 34896049 10.1016/j.joim.2021.11.006

[39] Zhang MM Zhao JW Li ZQ Shao J Gao XY. Acupuncture at Back-Shu point improves insomnia by reducing inflammation and inhibiting the ERK/NF-κB signaling pathway. World J Psychiatry. 2023;13:340–50. 37383281 10.5498/wjp.v13.i6.340 PMC10294136

[40] Zhao FY Fu QQ Kennedy GA. Can acupuncture improve objective sleep indices in patients with primary insomnia? A systematic review and meta-analysis. Sleep Med. 2021;80:244–59. 33610071 10.1016/j.sleep.2021.01.053

[41] Li X He F Tuo X. Electroacupuncture ameliorates peptic ulcer disease in association with gastroduodenal microbiota modulation in mice. Front Cell Infect Microbiol. 2022;12:935681. 36061878 10.3389/fcimb.2022.935681 PMC9437313

[42] Zhang L Chen X Wang H. “Adjusting internal organs and dredging channel” electroacupuncture ameliorates insulin resistance in type 2 diabetes mellitus by regulating the intestinal flora and inhibiting inflammation. Diabetes Metab Syndr Obes. 2021;14:2595–607. 34135611 10.2147/DMSO.S306861 PMC8200173

[43] Liu B Lin W Chen S. Gut microbiota as an objective measurement for auxiliary diagnosis of insomnia disorder. Front Microbiol. 2019;10:1770. 31456757 10.3389/fmicb.2019.01770 PMC6701205

[44] Feng Y Fu S Li C. Interaction of gut microbiota and brain function in patients with chronic insomnia: a regional homogeneity study. Front Neurosci. 2022;15:804843. 35069107 10.3389/fnins.2021.804843 PMC8766814

[45] Hong J Chen J Kan J Liu M Yang D. Effects of acupuncture treatment in reducing sleep disorder and gut microbiota alterations in pcpa-induced insomnia mice. Evid Based Complement Alternat Med. 2020;2020:3626120. 33178314 10.1155/2020/3626120 PMC7647758

[46] Dalile B Van Oudenhove L Vervliet B Verbeke K. The role of short-chain fatty acids in microbiota-gut-brain communication. Nat Rev Gastroenterol Hepatol. 2019;16:461–78. 31123355 10.1038/s41575-019-0157-3

[47] van de Wouw M Boehme M Lyte JM. Short-chain fatty acids: microbial metabolites that alleviate stress-induced brain-gut axis alterations. J Physiol. 2018;596:4923–44. 30066368 10.1113/JP276431 PMC6187046

[48] De Vadder F Kovatcheva-Datchary P Goncalves D. Microbiota-generated metabolites promote metabolic benefits via gut-brain neural circuits. Cell. 2014;156:84–96. 24412651 10.1016/j.cell.2013.12.016

[49] Han M Yuan S Zhang J. The interplay between sleep and gut microbiota. Brain Res Bull. 2022;180:131–46. 35032622 10.1016/j.brainresbull.2021.12.016

[50] Hua X Zhu J Yang T. The gut microbiota and associated metabolites are altered in sleep disorder of children with autism spectrum disorders. Front Psychiatry. 2020;11:855. 32982808 10.3389/fpsyt.2020.00855 PMC7493623

[51] Ouyang X Duan H Jin Q. Moxibustion may delay the aging process of Wistar rats by regulating intestinal microbiota. Biomed Pharmacother. 2022;146:112147. 34810050 10.1016/j.biopha.2021.112147

[52] Ke X Xiang Q Jiang P. Effect of electroacupuncture on short-chain fatty acids in peripheral blood after middle cerebral artery occlusion/reperfusion in rats based on gas chromatography-mass spectrometry. Mediators Inflamm. 2022;2022:3997947. 36052308 10.1155/2022/3997947 PMC9427317

[53] Zielinski MR Gibbons AJ. Neuroinflammation, sleep, and circadian rhythms. Front Cell Infect Microbiol. 2022;12:853096. 35392608 10.3389/fcimb.2022.853096 PMC8981587

[54] Chen HW Zhou R Cao BF. The predictive, preventive, and personalized medicine of insomnia: gut microbiota and inflammation. EPMA J. 2023;14:571–83. 38094575 10.1007/s13167-023-00345-1 PMC10713890

[55] Groschwitz KR Hogan SP. Intestinal barrier function: molecular regulation and disease pathogenesis. J Allergy Clin Immunol. 2009;124:3–20; quiz 21. 19560575 10.1016/j.jaci.2009.05.038 PMC4266989

[56] Li L. Research progress on infection microecology: the effect of intestinal microbiota on metabolism. Chin J Microecol. 2009;21:1–3.

[57] Shen N Wang Z Wang C Zhang J Liu C. Methane alleviates inflammation and apoptosis of dextran sulfate sodium-induced inflammatory bowel diseases by inhibiting toll-like receptor 4 (TLR4)/myeloid differentiation factor 88 (MyD88)/nuclear translocation of nuclear factor-κB (NF-κB) and endoplasmic reticulum stress pathways in mice. Med Sci Monit. 2020;26:e922248. 32500859 10.12659/MSM.922248 PMC7297035

[58] Su H Zhang C Zou X. Jiao-tai-wan inhibits inflammation of the gut-brain-axis and attenuates cognitive impairment in insomnic rats. J Ethnopharmacol. 2020;250:112478. 31843572 10.1016/j.jep.2019.112478

[59] Cai Y Gong D Xiang T Zhang X Pan J. Markers of intestinal barrier damage in patients with chronic insomnia disorder. Front Psychiatry. 2024;15:1373462. 38606411 10.3389/fpsyt.2024.1373462 PMC11007705

[60] Jang JH Yeom MJ Ahn S. Acupuncture inhibits neuroinflammation and gut microbial dysbiosis in a mouse model of Parkinson’s disease. Brain Behav Immun. 2020;89:641–55. 32827699 10.1016/j.bbi.2020.08.015

[61] Xu J Zheng X Cheng KK. NMR-based metabolomics reveals alterations of electro-acupuncture stimulations on chronic atrophic gastritis rats. Sci Rep. 2017;7:45580. 28358020 10.1038/srep45580 PMC5372362

[62] Xie DP Zhou GB Chen RL. Effect of electroacupuncture at zusanli (ST36) on sepsis induced by cecal ligation puncture and its relevance to spleen. Evid Based Complement Alternat Med. 2020;2020:1914031. 33082818 10.1155/2020/1914031 PMC7563055

[63] Li H Ye XF Su YS. Mechanism of acupuncture and moxibustion on promoting mucosal healing in ulcerative colitis. Chin J Integr Med. 2023;29:847–56. 35412218 10.1007/s11655-022-3531-x

[64] Bao C Wu L Wang D. Acupuncture improves the symptoms, intestinal microbiota, and inflammation of patients with mild to moderate Crohn’s disease: a randomized controlled trial. EClinicalMedicine. 2022;45:101300. 35198926 10.1016/j.eclinm.2022.101300 PMC8850329

[65] Bao CH Wang CY Li GN. Effect of mild moxibustion on intestinal microbiota and NLRP6 inflammasome signaling in rats with post-inflammatory irritable bowel syndrome. World J Gastroenterol. 2019;25:4696–714. 31528095 10.3748/wjg.v25.i32.4696 PMC6718040

[66] Dong ZQ Zhu J Lu DZ Chen Q Xu YL. Effect of electroacupuncture in “zusanli” and “kunlun” acupoints on TLR4 signaling pathway of adjuvant arthritis rats. Am J Ther. 2018;25:e314–9. 27574922 10.1097/MJT.0000000000000477

[67] Song G Fiocchi C Achkar JP. Acupuncture in inflammatory bowel disease. Inflamm Bowel Dis. 2019;25:1129–39. 30535303 10.1093/ibd/izy371

[68] Stilling RM Bordenstein SR Dinan TG Cryan JF. Friends with social benefits: host-microbe interactions as a driver of brain evolution and development? Front Cell Infect Microbiol. 2014;4:147. 25401092 10.3389/fcimb.2014.00147 PMC4212686

[69] Fidalgo S Ivanov DK Wood SH. Serotonin: from top to bottom. Biogerontology. 2013;14:21–45. 23100172 10.1007/s10522-012-9406-3

[70] Zhang H Liu P Wu X. Study on the effect of abdominal massage on hypothalamic activity and serum brain-gut peptide in patients with heart and spleen deficiency insomnia. Lishizhen Med Mater Med Res. 2022;33:397–400.

[71] Perry RJ Peng L Barry NA. Acetate mediates a microbiome-brain-β-cell axis to promote metabolic syndrome. Nature. 2016;534:213–7. 27279214 10.1038/nature18309 PMC4922538

[72] Cespuglio R. Serotonin: its place today in sleep preparation, triggering or maintenance. Sleep Med. 2018;49:31–9. 30029993 10.1016/j.sleep.2018.05.034

[73] Zhao J Tang Y Xia F. Research progress on the role of neurotransmitters in primary insomnia. Chin J Clin Neurosci. 2024;32:194–8.

[74] Agus A Planchais J Sokol H. Gut microbiota regulation of tryptophan metabolism in health and disease. Cell Host Microbe. 2018;23:716–24. 29902437 10.1016/j.chom.2018.05.003

[75] Ogawa Y Miyoshi C Obana N. Gut microbiota depletion by chronic antibiotic treatment alters the sleep/wake architecture and sleep EEG power spectra in mice. Sci Rep. 2020;10:19554. 33177599 10.1038/s41598-020-76562-9 PMC7659342

[76] Xu Z Li R Zhu C Li M. Effect of acupuncture treatment for weight loss on gut flora in patients with simple obesity. Acupunct Med. 2013;31:116–7. 22961606 10.1136/acupmed-2012-010209

[77] Luo B Wang Y Zhang Y. Effects of three acupuncture methods on the contents of 5-HT and 5-HIAA in hippocampus in PCPA insomnia rats. Chin J Basic Med Tradit Chin Med. 2016;22:1517–9.

[78] Chen C Zhou P Zhao B. Research progress on the relationship between brain-gut peptide ghrelin and brain dysfunction. Chin J Pract Diagn Therapy. 2019;33:1033–6.

[79] Burney BO Garcia JM. Hypogonadism in male cancer patients. J Cachexia Sarcopenia Muscle. 2012;3:149–55. 22528986 10.1007/s13539-012-0065-7 PMC3424192

[80] Denney WS Sonnenberg GE Carvajal-Gonzalez S Tuthill T Jackson VM. Pharmacokinetics and pharmacodynamics of PF-05190457: the first oral ghrelin receptor inverse agonist to be profiled in healthy subjects. Br J Clin Pharmacol. 2017;83:326–38. 27621150 10.1111/bcp.13127 PMC5237700

[81] Szentirmai E. Central but not systemic administration of ghrelin induces wakefulness in mice. PLoS One. 2012;7:e41172. 22815958 10.1371/journal.pone.0041172 PMC3398952

[82] Nilaweera KN Cabrera-Rubio R Speakman JR. Whey protein effects on energy balance link the intestinal mechanisms of energy absorption with adiposity and hypothalamic neuropeptide gene expression. Am J Physiol Endocrinol Metab. 2017;313:E1–E11. 28325732 10.1152/ajpendo.00356.2016

[83] Tubbs E Theurey P Vial G. Mitochondria-associated endoplasmic reticulum membrane (MAM) integrity is required for insulin signaling and is implicated in hepatic insulin resistance. Diabetes. 2014;63:3279–94. 24947355 10.2337/db13-1751

[84] Liu Q Cao J Liu M. TRPV1 was involved in the effects of different stimulation modes of acupuncture and moxibustion on ghrelin and GHSR-1α in mice. J Tradit Chin Med Univ Hunan. 2021;41:1487–92.

[85] Thomson F Craighead M. Innovative approaches for the treatment of depression: targeting the HPA axis. Neurochem Res. 2008;33:691–707. 17960478 10.1007/s11064-007-9518-3

[86] Misiak B Łoniewski I Marlicz W. The HPA axis dysregulation in severe mental illness: can we shift the blame to gut microbiota? Prog Neuropsychopharmacol Biol Psychiatry. 2020;102:109951. 32335265 10.1016/j.pnpbp.2020.109951

[87] Zangeneh FZ. Psychoneuroendocrinology aspect of sleep pattern in women with polycystic ovary syndrome. Annu Res Rev Biol. 2016;10:1–8.

[88] Buckley TM Schatzberg AF. On the interactions of the hypothalamic-pituitary-adrenal (HPA) axis and sleep: normal HPA axis activity and circadian rhythm, exemplary sleep disorders. J Clin Endocrinol Metab. 2005;90:3106–14. 15728214 10.1210/jc.2004-1056

[89] Vgontzas AN Bixler EO Lin HM. Chronic insomnia is associated with nyctohemeral activation of the hypothalamic-pituitary-adrenal axis: clinical implications. J Clin Endocrinol Metab. 2001;86:3787–94. 11502812 10.1210/jcem.86.8.7778

[90] Han X Gao Y Yin X. The mechanism of electroacupuncture for depression on basic research: a systematic review. Chin Med. 2021;16:10. 33436036 10.1186/s13020-020-00421-y PMC7805231

[91] Xiao A Wang H Liu H. Effect of heat-sensitive moxibustion on serum endocrine hormone levels in a rat model of insomnia. J Jiangxi Univ Tradit Chin Med. 2013;25:32–5.

[92] Jiao B Li Z Shi Y. Effect of electrical targeting on behavioral and HPA axis-related hormones in rats in a chronic stress model. Acupunct Res. 2004;4:252–6.

[93] Xie C Wang J Zhao N. Effects of electroacupuncture on sleep via the dopamine system of the HPA axis in rats after cage change. Evid Based Complement Alternat Med. 2021;2021:5527060. 34306138 10.1155/2021/5527060 PMC8270700

[94] Lv Z Liu R Su K. Acupuncture ameliorates breast cancer-related fatigue by regulating the gut microbiota-gut-brain axis. Front Endocrinol (Lausanne). 2022;13:921119. 36093113 10.3389/fendo.2022.921119 PMC9449876

[95] Henao-Mejia J Strowig T Flavell RA. Microbiota keep the intestinal clock ticking. Cell. 2013;153:741–3. 23663774 10.1016/j.cell.2013.04.043

[96] Ait-Belgnaoui A Durand H Cartier C. Prevention of gut leakiness by a probiotic treatment leads to attenuated HPA response to an acute psychological stress in rats. Psychoneuroendocrinology. 2012;37:1885–95. 22541937 10.1016/j.psyneuen.2012.03.024

[97] Xie LL Zhao YL Yang J. Electroacupuncture prevents osteoarthritis of high-fat diet-induced obese rats. Biomed Res Int. 2020;2020:9380965. 32724821 10.1155/2020/9380965 PMC7366230

[98] Wang JM Yang MX Wu QF. Improvement of intestinal flora: accompany with the antihypertensive effect of electroacupuncture on stage 1 hypertension. Chin Med. 2021;16:7. 33413552 10.1186/s13020-020-00417-8 PMC7792359

[99] Wang Z Wang Z Lu T. The microbiota-gut-brain axis in sleep disorders. Sleep Med Rev. 2022;65:101691. 36099873 10.1016/j.smrv.2022.101691

[100] Crunkhorn S. Understanding GABAA receptor pharmacology. Nat Rev Drug Discov. 2023;22:873. 10.1038/d41573-023-00162-1 37798464

[101] Bravo JA Forsythe P Chew MV. Ingestion of Lactobacillus strain regulates emotional behavior and central GABA receptor expression in a mouse via the vagus nerve. Proc Natl Acad Sci USA. 2011;108:16050–5. 21876150 10.1073/pnas.1102999108 PMC3179073

[102] Zhang F Zhang X Peng Q Tang L. Electroacupuncture of the cymba concha alleviates p-chlorophenylalanine-induced insomnia in mice. Acupunct Med. 2023;41:345–53. 37081732 10.1177/09645284231160193

[103] Gong R Liu X Zhao J. Electroacupuncture-induced activation of GABAergic system alleviates airway inflammation in asthma model by suppressing TLR4/MyD88/NF-κB signaling pathway. Chin Med J (Engl). 2023;136:451–60. 36867547 10.1097/CM9.0000000000002314 PMC10106183

[104] Ruan J. Effects of electro-acupuncture on the content of glutamate and gamma-aminobutyric acid and the expression of gamma-aminobutyric acid type A receptor in the brain of insomnia rats. China J Tradit Chin Med Pharm. 2013;28:3657–60.

[105] Xiao M Wang XS He C Huang ZS Chen HR Kong LH. The gut-brain axis: effect of electroacupuncture pretreatment on learning, memory, and JNK signaling in D-galactose-induced AD-like rats. Iran J Basic Med Sci. 2023;26:532–9. 37051108 10.22038/IJBMS.2023.66954.14683 PMC10083831

